# Protective Effects of an Ancient Chinese Kidney-Tonifying Formula against H_2_O_2_-Induced Oxidative Damage to MES23.5 Cells

**DOI:** 10.1155/2017/2879495

**Published:** 2017-03-12

**Authors:** Yihui Xu, Wei Lin, Shuifen Ye, Huajin Wang, Tingting Wang, Youyan Su, Liangning Wu, Yuanwang Wang, Qian Xu, Chuanshan Xu, Jing Cai

**Affiliations:** ^1^Second People's Hospital, Fujian University of Traditional Chinese Medicine, Fuzhou, Fujian Province 350003, China; ^2^Academy of Integrative Medicine, Fujian University of Traditional Chinese Medicine, Fuzhou, Fujian Province 350122, China; ^3^Longyan First Hospital Affiliated to Fujian Medical University, Longyan, Fujian Province 364000, China; ^4^Tibet Autonomous Region People's Hospital, Lhasa, Tibet Autonomous Region 850000, China; ^5^College of Integrative Medicine, Fujian University of Traditional Chinese Medicine, Fuzhou, Fujian Province 350122, China; ^6^Graduate School, Fujian University of Traditional Chinese Medicine, Fuzhou, Fujian Province 350122, China; ^7^Quanzhou Orthopedic-Traumatological Hospital, Fujian University of Traditional Chinese Medicine, Quanzhou, Fujian Province 362000, China; ^8^School of Chinese Medicine, Faculty of Medicine, The Chinese University of Hong Kong, Shatin, Hong Kong

## Abstract

Oxidative damage plays a critical role in the etiology of neurodegenerative disorders including Parkinson's disease (PD). In our study, an ancient Chinese kidney-tonifying formula, which consists of* Cistanche*,* Epimedii,* and* Polygonatum cirrhifolium*, was investigated to protect MES23.5 dopaminergic neurons against hydrogen peroxide- (H_2_O_2_-) induced oxidative damage. The damage effects of H_2_O_2_ on MES23.5 cells and the protective effects of KTF against oxidative stress were evaluated using MTT assay, transmission electron microscopy (TEM), immunocytochemistry (ICC), enzyme-linked immunosorbent assay (ELISA), and immunoblotting. The results showed that cell viability was dramatically decreased after a 12 h exposure to 150 *μ*M H_2_O_2_. TEM observation found that the H_2_O_2_-treated MES23.5 cells presented cellular organelle damage. However, when cells were incubated with KTF (3.125, 6.25, and 12.5 *μ*g/ml) for 24 h after H_2_O_2_ exposure, a significant protective effect against H_2_O_2_-induced damage was observed in MES23.5 cells. Using ICC, we found that KTF inhibited the reduction of the tyrosine hydroxylase (TH) induced by H_2_O_2_, upregulated the mRNA and protein expression of HO-1, CAT, and GPx-1, and downregulated the expression of caspase 3. These results indicated that KTF may provide neuron protection against H_2_O_2_-induced cell damage through ameliorating oxidative stress, and our findings provide a new potential strategy for the prevention and treatment of Parkinson's disease.

## 1. Introduction

Neurodegenerative diseases have recently become an important public health problem. Parkinson's disease (PD) is a neurodegenerative disease characterized by the progressive loss of dopaminergic neurons in the substantia nigra. Even though the cause of PD remains to be clarified, several lines of evidence strongly suggest that oxidative damage and mitochondrial dysfunction play an important role in the pathological mechanisms of PD [1–3]. Increased oxidative stress results from imbalance between oxidative products such as reactive oxygen species (ROS) and antioxidant molecules in the cell [4]. Enhancing antioxidative capacity of brain cells has become an effective strategy in protecting nervous cells against oxidative stress damage.

PD is a difficult-to-treat disease threatening the elderly worldwide. The current therapeutic drugs have limited efficacy with severe side-effects. Traditional Chinese herbs and formula have been widely used in folk medicine to treat neurodegenerative diseases including PD for a long time. According to Chinese medicine theory, the deficiency of kidney is the main pathological mechanism of PD.* Cistanche deserticola* Y. C. Ma, a species of* Cistanche* which belongs to the Orobanchaceae family, is a well-known herb in traditional Chinese medicine with so-called kidney-tonifying efficacy and is used for the treatment of kidney deficiency, female infertility, morbid leucorrhea, neurasthenia, and senile constipation [5]. It has been reported that* Cistanche* total glycosides exerted protective effects on substantia nigra dopaminergic neuron in mice model and cells of PD [6, 7]. Moreover, the active ingredients of* Cistanche* have been documented to possess neuroprotective effect through enhancing antioxidant capacity and inhibiting apoptosis of neurons [8–15].* Herba Epimedii* is also a traditional kidney-tonifying Chinese herb with significantly antioxidative activity, which is widely utilized in treating osteoporosis and cardiovascular diseases and improving sexual and neurological functions [16].* Polygonatum sibiricum* is often employed as an assistant to improve the effectiveness of the monarch drug and minister drug in the traditional Chinese formula. Recent report showed that the water extracts of* Polygonatum sibiricum* are natural antioxidants.

Based on these benefits from the ancient Chinese kidney-tonifying formula (KTF) which consists of* Cistanche*,* Epimedii*, and* Polygonatum cirrhifolium*, we hypothesized that KTF might protect MES23.5 dopaminergic neurons against hydrogen peroxide- (H_2_O_2_-) induced oxidative damage. Herein, as proof-of-concept, we conducted a series of experiments to examine the effects of KTF on cell death and antioxidant enzymes expression in the H_2_O_2_-treated MES23.5 cells.

## 2. Materials and Methods

### 2.1. Chemicals and Reagents

HO-1, CAT, caspase 3, and *β*-actin antibodies were obtained from Cell Signaling Technology Inc. (Boston, USA). GPx-1 antibody was from Abcam (Cambridge Science Park, UK). Rabbit Anti-Tyrosine Hydroxylase was from Boster (BA1454, China). Goat anti-rabbit IgG1 conjugated to a horseradish peroxidase label was from Santa Cruz Biotechnology Inc. (California, USA). Dulbecco's modified Eagle's medium (DMEM) was purchased from GIBCO BRL Co. Ltd. (Gaithersburg, MD, USA). All the other reagents, unless otherwise stated, were from Sigma Chemical Co. (St. Louis, MO, USA). RevertAid™ First Strand cDNA Synthesis Kit was from Fermentas Inc. (Burlington, USA). DreamTaq Green PCR Master Mix (2x) was from Fermentas Inc. (Burlington, USA). Bio DL100 was from BioFlux (Tokyo, Japan). Polink-2 plus Polymer HRP Detection System for Rabbit Primary Antibody (PV-9001) and DAB (3, 3′-diaminoobenzidine tetrahydrochloride) (ZLI-9032) were from ZSGB-BIO (Beijing, China). ELISA kits for measuring CAT, GPx-1, and GSH were from Hufeng Biotechnology Inc. (Shanghai, China).* Ginkgo biloba* extracts (GE) were from Shanghai Sine Promod Pharmaceutical Co., Ltd. (Shanghai, China).

### 2.2. Preparation and Standardization of Kidney-Tonifying Formula

The components of kidney-tonifying formula*, Cistanche deserticola*,* Epimedium brevicornum, *and* Polygonatum sibiricum*, were supplied by Second Affiliated People's Hospital, Fujian University of Traditional Chinese Medicine, and carefully authenticated according to the Chinese pharmacopoeia (The Pharmacopoeia Commission of China, 2005). To prepare KTF samples,* Cistanche deserticola*,* Epimedium brevicornum, *and* Polygonatum sibiricum* at the weight ratio of 1 : 1 : 1.2 were mixed and ground into the powder and then immersed in a total volume of 10 times (w/v) distilled water for 1 h and boiled for 2 h. After the solution was filtered, the filtrate was collected. The entire residue was collected and further boiled with a total volume of 8 times (w/v) distilled water for 2 h. The solution was filtered and the two filtrates were combined, concentrated, and freeze-dried. The yield of the final dried extract was 25% (w/w) of the starting raw herbal materials and was stored at −20°C until used. The stock solution KTF (10 mg/ml) was prepared by dissolving KTF in phosphate-buffer solution (PBS), followed by sonication, sterilization at 100°C, and filtration.

### 2.3. Cell Culture and H_2_O_2_ Treatment

MES23.5 cells were cultured and exposed to H_2_O_2_ as described by our previous paper [10]. Briefly, MES23.5 cells were grown in DMEM/F12 (GIBCO BRL Co. Ltd., Gaithersburg, MD, USA) medium containing 5% (vol/vol) FBS (GIBCO BRL Co. Ltd., Gaithersburg, MD, USA), 100 U penicillin/streptomycin, 2 mM L-glutamine (Sigma Chemical Co., St. Louis, MO, USA), and Sato's (50 × Sato's: insulin 25 mg; transferring 25 mg; pyruvic acid, 243 mg; putrescine 20 mg; 1 mg/ml sodium selenate, 25 *μ*l; 0.315 mg/ml progesterone) at 37°C with 5% CO_2_. Overnight grown MES23.5 cells were further incubated with 150 *μ*M H_2_O_2_ for 12 h.

### 2.4. KTF Treatment

MES23.5 cells were randomly divided into control group (fresh culture medium), H_2_O_2_ group (H_2_O_2_ culture medium),* Ginkgo biloba* extract (GE) group (H_2_O_2_ and GE culture medium), and KTF groups including low dose of KTF treatment, middle dose of FTF treatment, and high dose of KTF treatment. MES23.5 cells at logarithmic phase were seeded in culture plate coated by poly-L-lysine (PLL) for 24 h. The cells in the control group were treated with the fresh culture medium, and those in all other groups were treated with the culture medium containing 150 *μ*M H_2_O_2_ for 12 h. The cells in the GE group were treated with the culture medium containing 12.5 *μ*g/ml GE, and those in the low dose KTF group, middle dose KTF group, and high dose KTF group were treated with KTF at the concentrations of 3.125, 6.25, and 12.5 *μ*g/ml for 24 h, respectively.

### 2.5. Cell Viability

After KTF treatment for 20 h, the cells were incubated with MTT (0.5 mg/ml) for 4 h at 37°C, and the media were carefully removed and 150 *μ*L of DMSO was added to each well. The formazan crystals were dissolved for 10 min, and absorbance was measured at 490 nm. Optical density (OD) of each well was measured by spectrophotometer (BIO-TEK ELX 800, BioTek Instruments Inc., Vermont, USA). Freshly cell culture medium was used as a negative control.

### 2.6. Ultrastructural Morphology Observation

After KTF treatment for 24 h, the cells were collected, suspended, and fixed with 3% glutaraldehyde in 1.5% paraformaldehyde solution (pH 7.3) at 4°C for 24 h. Cell suspensions were then rinsed twice with PBS and postfixed with 1% osmic acid and 1.5% potassium hexacyanoferrate (II) solution (pH 7.3) and incubated at 4°C for 2 h; then cell suspensions were dehydrated in a graded series of alcohol for 5 min each. The dehydrated pellets were embedded three times with propylene oxide for 1 h and infiltrated with a resin/propylene oxide mixture at 1 : 1 ratio for 2 h and then with resin for 12 h at room temperature. The inclusion was made with epoxy resin 618 and Araldite and polymerization was performed at 60°C for 48 h. Finally, ultrathin sections (80 nm) were stained with uranyl acetate and counterstained with lead citrate for 5 min. The stained cells were examined with the H7650 transmission electron microscope (Hitachi, Japan).

### 2.7. Immunocytochemical Assay of Tyrosine Hydroxylase (TH)

After KTF treatment for 24 h, the cells were washed twice for 2 min with PBS and fixed with 4% paraformaldehyde for 15 min. The fixed cells were washed 3 times and then treated with 0.5% Triton X-100 for 20 min. Afterward, the cells were washed 3 times and treated with 3% H_2_O_2_ for 10 min. The H_2_O_2_-treated cells were washed 3 times and then blotted with goat serum for 20 min. After being blotted, rabbit anti-TH (1 : 400) was added to each sample at 4°C overnight. The samples were then washed 3 times and treated with Polymer Helper for 20 min. After Polymer treatment the cells were washed 3 times and treated with poly-HRP anti-rabbit IgG for 20 min. The cells were washed 3 times and treated with DAB for 3 min. Finally, the samples were washed with running water and treated with hematoxylin counterstaining for 1 min and then washed with running water and sealed with permount mounting medium.

### 2.8. Measurement of Antioxidative Enzymes by ELISA

After KTF treatment for 24 h, the activity level of intracellular CAT, GPx-1, and GSH was measured using an ELISA kit (Hufeng Biotechnology Inc., Shanghai, China) according to the manufacturer's instruction. Briefly, 100 *μ*L homogenate supernatant of the lysate of the treated cells was added to the ELISA wells and then incubated at 37°C for 30 min. After being washed five times and dried, 50 *μ*L enzyme-labeled antibody working solution was added to each well (expected control tube). The resultant solution was incubated for 30 min at 37°C and washed five times and dried. Substrate working solution (150 *μ*L) was added to each well and incubated for 15 min at 37°C. Finally, stop solution (50 *μ*L) was added to each well and the absorbance value was determined at 450 nm using an automatic ELISA reader (XL800, BioTex, Incorporated, Houston, TX, USA).

### 2.9. Western Blot Analysis

After KTF treatment for 24 h, the cells were washed with ice-cold PBS and proteins were extracted in a lysis buffer containing 50 mM Tris-HCl (pH 7.4), 1 mM EDTA, 150 mM NaCl, 1% Nonidet P-40, 1 mM phenylmethylsulfonyl fluoride, and 10 *μ*g/mL aprotinin. Protein concentration was determined using BCA Protein Assay Kit (Thermo Scientific Pierce, Rockford, Illinois, USA). Protein were separated by SDS-PAGE electrophoresis and transferred to PVDF (Bio-Rad, Hercules, CA, USA). Blocking was performed with 5% nonfat dried milk; the membrane was incubated with the of rabbit anti-rat HO-1, CAT, caspase 3, *β*-actin antibody (1 : 1000, Cell Signaling), and rabbit anti-rat GPx-1 antibody (1 : 1000, Abcam) overnight at 4°C. The treated membrane was washed and then exposed to goat anti-rabbit IgG1 conjugated to a horseradish peroxidase label (1 : 5000, Santa Cruz, USA) for 2 h at room temperature. After five more washes, cross-reactivity was visualized using Electrochemiluminescence (ECL) Western blotting detection reagents and analyzed using scanning densitometry in an Image System (Bio-Rad, Hercules, CA, USA).

### 2.10. RT-PCR Analysis

PCR primer of HO-1, CAT, GPx-1, and *β*-actin were purchased from Sangon Biotech Co. Ltd. (Shanghai, China). After KTF treatment for 24 h, total RNA of cells was isolated using Trizol reagent according to the manufacturer's instruction. Reverse transcription (RT) was performed using the AMV reverse transcription system (Fermentas, Burlington, USA). cDNA fragment and *β*-actin were amplified using Green PCR master MIX Kit (Fermentas, Burlington, USA). DNA was amplified immediately with a single cycle at 94°C for 5 min and 30 cycles at 94°C for 30 s and 58°C for 30 s and 72°C for 30 s for *β*-actin, and a final extension step was taken at 72°C for 10 min. Ethidium bromide stained gels were scanned and qualified using Tanon Image Software. The intensity of each band was normalized against the intensity of *β*-actin.

### 2.11. Statistical Analysis

Statistical analysis was done with SPSS 18.0 (SPSS Inc., Chicago, IL). All data were shown as mean ± SD (standard deviation), and statistical significance of difference was performed by one-way ANOVA and LSD (linear standard deviation). A difference was considered to be significant at *P* < 0.05.

## 3. Results

### 3.1. The Damage Effects of H_2_O_2_ on MES23.5 Cells and the Protective Effects of KTF against Oxidative Stress

**Figure 1:**
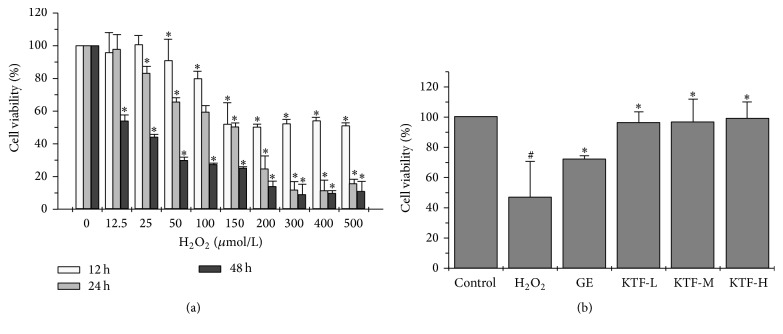
The damage effects of H_2_O_2_ on MES23.5 cells and the protective effects of KTF against oxidative stress were evaluated using MTT assay. (a) Effects of different concentrations of H_2_O_2_ treatment on cell damage. MES23.5 cells were treated with 12.5, 25, 50, 100, 150, 200, 300, 400, and 500 *μ*Mol/L H_2_O_2_ for 12, 24, and 48 h. (b) Protective effects of different concentrations of KTF treatment on H_2_O_2_-induced cell damage. Treatment of KTF resulted in increase of MES23.5 cells viability after H_2_O_2_ addition. MES23.5 cells were incubated with GE (12.5 *μ*g/ml) or KTF (3.125, 6.25, 12.5 *μ*g/ml) for 24 h after the addition of H_2_O_2_. The cell viability was measured with MTT. Data are expressed as mean ± SD (standard deviation) in three independent experiments. ^#^*P* < 0.05 compared to the control group. ^*∗*^*P* < 0.05 compared to the H_2_O_2_-treat group.

As shown in Figure 1(a), H_2_O_2_ exhibited a dose- and time-dependent inhibition in the viability of MES23.5 dopaminergic neuronal cells. After 12 h exposure to 50 *μ*M H_2_O_2_, a significant loss of cell viability was noted, and the cell viability loss gradually increased with H_2_O_2_ dose increasing to 150 *μ*M. However, further increasing H_2_O_2_ dose from 200 to 500 *μ*M did not result in significant higher loss of cell viability. Extending MES23.5 cells exposure to H_2_O_2_ from 12 h to 24 h and 48 h increased the loss of cell viability at lower H_2_O_2_ concentration. A significant loss of MES23.5 cell viability was noted after 24 h exposure to 25 *μ*M H_2_O_2_ and 48 h exposure to 12.5 *μ*M H_2_O_2_. A time-dependent manner of cell viability loss was observed in all tested concentration of H_2_O_2_.

Exposing MES23.5 cells to KTF alone did not have significant cytotoxic effect even when KTF concentration was up to 100 *μ*g/ml. MES23.5 cells cocultured with 150 *μ*M H_2_O_2_ for 12 h caused cell viability loss of 46.76%. Cytotoxic effect of H_2_O_2_ on MES23.5 cells was significantly attenuated upon KTF treatment for 24 h (Figure 1(b)). Augmented viability of H_2_O_2_ treated MES23.5 cells was restored to 95.86%  ± 7.18%, 96.49%  ± 15.07%, and 99.04%  ± 10.57% by a treatment with KTF at concentrations of 3.125, 6.25, and 12.5 *μ*g/ml, respectively.

**Figure 2:**
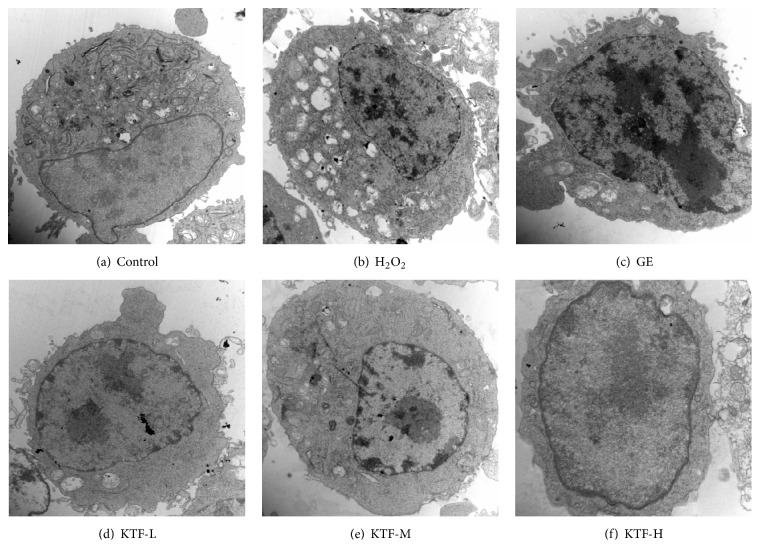
Effect of KTF on cellular ultrastructure in H_2_O_2_-induced MES23.5 cells. Cellular ultrastructure was observed under TEM. Normal morphology of cytoplasm, cell organelles in control group ((a), ×15,000), characteristic ultrastructural morphology of oxidative damage in H_2_O_2_-treated group ((b), ×15,000), treatment with KTF group (3.125, 6.25, and 12.5 *μ*g/ml, respectively) ((d), (e), (f), ×15,000), or* Ginkgo* extract (12.5 *μ*g/ml) ((c), ×15,000).

TEM showed that normal cells in control group exhibited oval shape with intact cell membrane which has many corrugations and microvilli-like structures. Cell nucleus was elliptic in the center with a single nucleolus and had normal euchromatin and scattered heterochromatin. Mitochondria exhibited rod-like or elliptic, scattered, and with normal cristae mitochondriate. Rough endoplasmic reticulum was well developed and had no obvious degranulation (Figure 2(a)). However, the cells treated with H_2_O_2_ presented typical oxidative damage with smaller, irregular nucleus, and swelled mitochondria. Obvious degranulation was found in rough endoplasmic reticulum (Figure 2(b)). In the cells treated by KTF ameliorated H_2_O_2_-induced oxidative damage, mitochondria exhibited rod-like or elliptic, scattered, and with more cristae mitochondriate. Rough endoplasmic reticulum had no obvious degranulation (Figures 2(d), 2(e), and 2(f)).

### 3.2. Increasing Expression of TH in MES23.5 Cells after KTF Treatment

**Figure 3:**
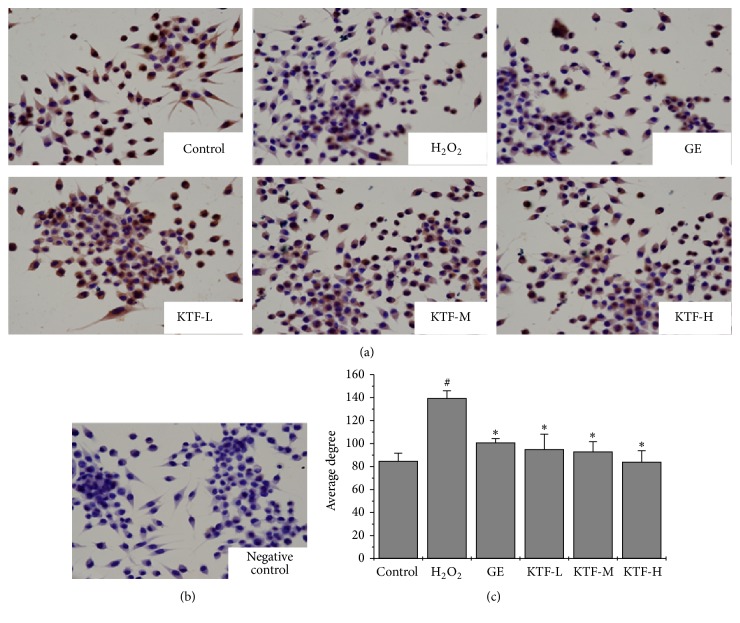
KTF ameliorated H_2_O_2_-induced reduction of TH in MES23.5 cells. Cells were fixed with 4% paraformaldehyde and the expression of TH was detected by immunocytochemistry. (a) Tan represents the TH positive expression (×400). (b) Negative control. (c) Results are expressed as average grey degree. Data are mean ± SD in three independent experiments. ^#^*P* < 0.05 compared to the control group. ^*∗*^*P* < 0.05 compared to the H_2_O_2_-treat group.

TH is a critical catalyzing enzyme in the conversion of the amino acid L-tyrosine to L-3,4-dihydroxyphenylalanine (L-DOPA) which is the precursor to the neurotransmitters dopamine. The normal expression of TH is the mark of active neuron. Therefore, we compared the TH expression in the H_2_O_2_-incubated MES23.5 cells with or without following KTF treatment by immunohistochemistry. The result showed that H_2_O_2_ decreased TH expression in the MES23.5 cells, and following KTF treatment could improve TH expression significantly (Figure 3). This indicates that KTF treatment was able to restore the function of the H_2_O_2_-treated MES23.5 cells.

### 3.3. KTF Suppressed H_2_O_2_-Induced Caspase 3 Activation in MES23.5 Cells

**Figure 4:**
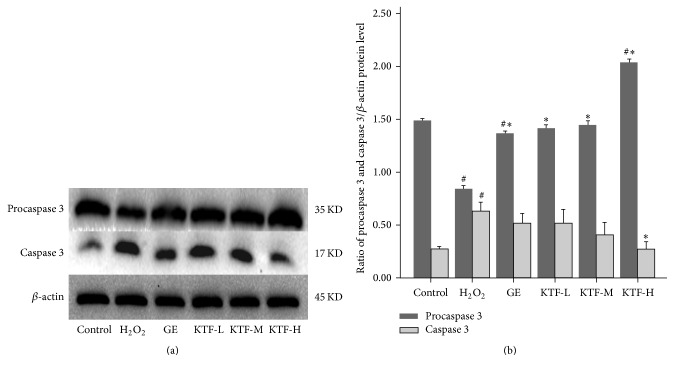
Effect of KTF on the protein expression of caspase 3 in H_2_O_2_-induced MES23.5 cells. (a) The protein expression of caspase 3 was determined with western blot and quantified in (b). Data are expressed as mean ± SD in three independent experiments. ^#^*P* < 0.05 compared to the control group. ^*∗*^*P* < 0.05 compared to the H_2_O_2_ -treat group.

For further investigating the mechanism of MES23.5 cell damage, we evaluated the expression of proapoptosis factor caspase 3 by immunoblotting. Result showed that H_2_O_2_ treatment markedly downregulated the expression of procaspase 3 and upregulated the expression of caspase 3. In contrast, KTF treatment restored the procaspase 3 and caspase 3 balance by increasing the expressions of procaspase 3 and decreasing the expression of caspase 3 (Figure 4). These findings demonstrate that KTF treatment effectively inhibited caspase 3 activation.

### 3.4. Increased Expression of Antioxidative Enzymes in MES23.5 Cells by KTF Treatment

**Figure 5:**
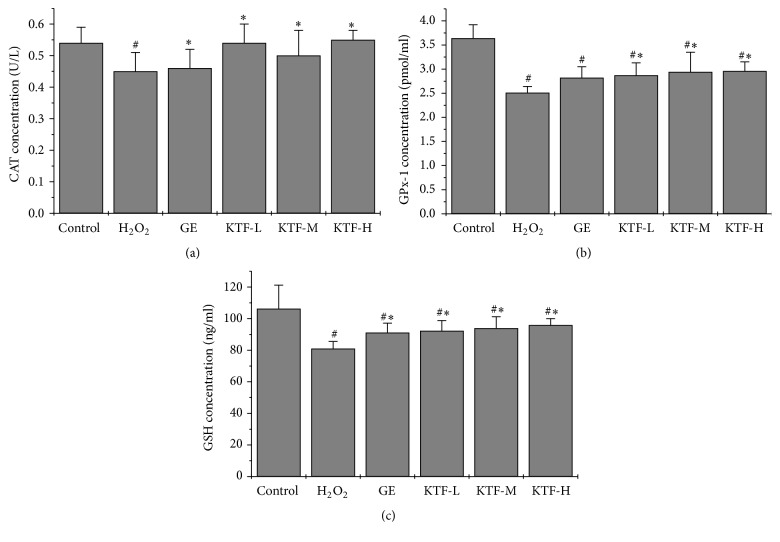
Effect of KTF on the level of intracellular CAT, GPx-1, and GSH in H_2_O_2_-induced MES23.5 cells. The activity level of intracellular CAT, GPx-1, and GSH was measured using an ELISA kit. Data are expressed as mean ± SD in three independent experiments. ^#^*P* < 0.05 compared to the control group. ^*∗*^*P* < 0.05 compared to the H_2_O_2_-treat group.

**Figure 6:**
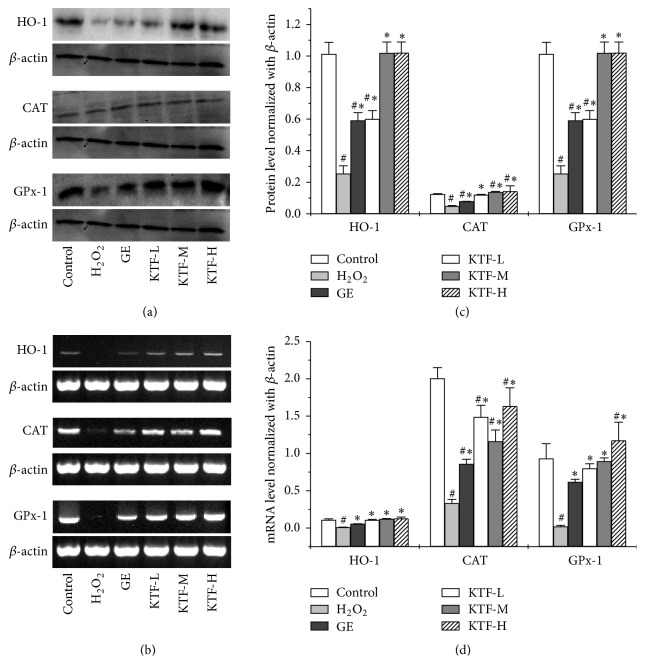
KTF increased the protein and mRNA expression of antioxidant enzymes in H_2_O_2_-induced MES23.5 cells. (a) The protein expressions of HO-1, CAT, and GPx-1 were determined with Western blot and quantified in (c). (b) The mRNA expressions of HO-1, CAT, and GPx-1 were determined with RT-PCR and quantified in (d). Data are expressed as mean ± SD in three independent experiments. ^#^*P* < 0.05 compared to the control group. ^*∗*^*P* < 0.05 compared to the H_2_O_2_ -treat group.

ELISA assay revealed that intracellular CAT, GPx-1, and GSH were downregulated after 150 *μ*M H_2_O_2_ treatment in MES23.5 cells. Of note, following treatment of KTF restored intracellular CAT, GPx-1, and GSH expression. RT-PCR analysis indicated that H_2_O_2_ treatment downregulated CAT and GPx-1 mRNA expression; however, KTF treatment restored the expressions of CAT and GPx-1 mRNA in MES23.5 cells. Immunoblot with specific antibodies also confirmed that CAT and GPx-1 protein expression in MES23.5 cells were downregulated by H_2_O_2_ treatment, and KTF treatment was able to restore CAT and GPx-1 protein expression. Moreover, mRNA expression of oxidative stress responding protein HO-1 was also inhibited after H_2_O_2_ treatment and then was restored by following KTF treatment. Immunoblotting also proved that HO-1 protein expression was markedly downregulated by H_2_O_2_, but KTF treatment was able to restore HO-1 protein expression. As expected, the highest KTF concentration used in our investigation has the best effect on restoring CAT, GPx-1, GSH, and HO-1 expression (Figures 5 and 6). Taken together, the obtained results demonstrate that KTF treatment significantly upregulated antioxidant substrates in the H_2_O_2_-treated MES23.3 cells.

## 4. Discussion

As an important component of redox system, ROS play important roles in regulating cell function. Moderate levels of ROS may function as signals to promote cell proliferation and survival. Under physiologic conditions, the balance between generation and elimination of ROS maintains the proper function of redox-sensitive signaling system. Redox homeostasis can be disrupted by increase of ROS production or decrease of ROS-scavenging capacity, leading to an overall increase in oxidative stress [17, 18], subsequently causing an irreversible damage of intracellular structure and inducing cell death.

High consumption of O_2_ in brain leads to accumulation of ROS; thus, neurons are particularly vulnerable to the attack of oxidative stress. Postmortem investigations have shown lipid peroxidation and oxidative damage to brain cells in PD patients [19]. Growing studies confirm that oxidative damage plays a critical role in the pathogenesis of neuron degenerative diseases, such as AD, PD, HD, and ALS [18, 20]. In the present study, a significant loss of cell viability was observed in MES23.5 dopaminergic neuronal cell after exposure to 50 *μ*M H_2_O_2_ for 12 h, 25 *μ*M H_2_O_2_ for 24 h, or 12.5 *μ*M H_2_O_2_ for 48 h. As shown by morphology observation, the cells exposed 12 h to 150 *μ*M H_2_O_2_ exhibited a typical oxidative injury including multiple membranous vacuoles in the cytoplasm, disruption of endoplasmic reticulum and mitochondrion, and condensing euchromatin in cell nucleus.

According to Chinese medicine theory, the deficiency of kidney is the main pathological mechanism of PD. Invigorating the kidney is an important strategy in the treatment of PD. In the present study, we found that traditional kidney-tonifying formula KTF, which consists of* Cistanche*,* Epimedii,* and* Polygonatum sibiricum*, significantly enhanced cell viability and the expression of TH of H_2_O_2_-treated MES23.5 cells.

Cell apoptosis is a mode of programmed cell death upon various stimuli which activate either extrinsic or intrinsic pathways. Cysteinyl aspartate-specific proteases (caspases) are highly conserved in multicellular organisms and function as central regulators of apoptosis. Activation of caspases is the main axis of all apoptosis events. Among those caspases, caspase 3 has been identified as a key mediator and indicator of cell apoptosis. Like other caspases, caspase 3 usually exists as an inactive proenzyme (precaspase 3). After proteolytic processing, it will produce two subunits which can dimerize to form active caspase 3, subsequently evoking many of the biochemical and biophysical changes to induce cell apoptosis [21]. Our present study revealed that H_2_O_2_ treatment significantly upregulated caspase 3, but this trend was reversed by KTF treatment in MES23.5 cells, indicating that KTF reduced cell apoptosis of H_2_O_2_-treated MES23.5 cells.

Antioxidative enzymes, such as superoxide dismutase (SOD), CAT, GPx-1, GSH, and HO-1, are important defense mechanism of cells against the oxidative stress. As an antioxidative enzyme, SOD can transform superoxide radicals to hydrogen peroxide which was subsequently converted to H_2_O by CAT and GPx [22]. GSH is also an important antioxidant. Catalytic conversion of reduced glutathione (GSH) to oxidized glutathione (GSSG) by GPx is helpful to convert H_2_O_2_ to O_2_. It has been reported that the activity of the antioxidant was decreased in PD model mice [23–25]. Therefore, drugs that regulate expression of enzymatic antioxidants may serve as potential candidates for the treatment of PD. Our data showed that H_2_O_2_ treatment suppressed CAT, GPx, GSH, and HO-1 in MES23.5 cells, while KTF treatment at different concentration effectively restored the expression of CAT, GPx, GSH, and HO-1.

In conclusion, our findings demonstrate that traditional Chinese herb kidney-tonifying formulation, which consists of* Cistanche*,* Epimedii,* and* Polygonatum sibiricum*, has strong antioxidative capacity and significant neuroprotective effects. This study provides a clue for developing the kidney-tonifying formulation as a potential therapeutic agent to prevent and treat Parkinson's disease.
